# AAV-mediated cardiac gene transfer of wild-type desmin in mouse models for recessive desminopathies

**DOI:** 10.1038/s41434-020-0147-7

**Published:** 2020-04-22

**Authors:** T. Ruppert, M. B. Heckmann, K. Rapti, D. Schultheis, A. Jungmann, H. A. Katus, L. Winter, N. Frey, C. S. Clemen, R. Schröder, O. J. Müller

**Affiliations:** 1 Department of Internal Medicine III, University of Kiel, Kiel, Germany; 2 Internal Medicine III, University Hospital Heidelberg, Heidelberg, Germany; 3 DZHK (German Centre for Cardiovascular Research), Partner Site Hamburg/Kiel/Lübeck, Kiel, Germany; 4 DZHK (German Center for Cardiovascular Research), Partner Site Heidelberg/Mannheim, Heidelberg, Germany; 5 Institute of Neuropathology, University Hospital Erlangen, Friedrich-Alexander University Erlangen-Nürnberg, Erlangen, Germany; 6 Department of Cell- and Developmental Biology, Center for Anatomy and Cell Biology, Medical University of Vienna, Vienna, Austria; 7 Institute of Aerospace Medicine, German Aerospace Center (DLR), Cologne, Germany; 8 Center for Physiology and Pathophysiology, Institute of Vegetative Physiology, Medical Faculty, University of Cologne, Cologne, Germany; 9 Muscle Research Center Erlangen (MURCE), Friedrich-Alexander-Universität Erlangen-Nürnberg, Erlangen, Germany

**Keywords:** Cardiovascular diseases, Gene therapy

## Abstract

Mutations in the human desmin gene cause autosomal-dominant and recessive cardiomyopathies and myopathies with marked phenotypic variability. Here, we investigated the effects of adeno-associated virus (AAV)-mediated cardiac wild-type desmin expression in homozygous desmin knockout (DKO) and homozygous R349P desmin knockin (DKI) mice. These mice serve as disease models for two subforms of autosomal-recessive desminopathies, the former for the one with a complete lack of desmin protein and the latter for the one with solely mutant desmin protein expression in conjunction with protein aggregation pathology in striated muscle. Two-month-old mice were injected with either a single dose of 5 × 10^12^ AAV9-hTNT2-mDes (AAV-Des) vector genomes or NaCl as control. One week after injection, mice were subjected to a forced swimming exercise protocol for 4 weeks. Cardiac function was monitored over a period of 15 month after injection and before the mice were sacrificed for biochemical and morphological analysis. AAV-mediated cardiac expression of wild-type desmin in both the homozygous DKO and DKI backgrounds reached levels seen in wild-type mice. Notably, AAV-Des treated DKO mice showed a regular subcellular distribution of desmin as well as a normalization of functional and morphological cardiac parameters. Treated DKI mice, however, showed an aberrant subcellular localization of desmin, unchanged functional cardiac parameters, and a trend toward an increased cardiac fibrosis. In conclusion, the effect of a high-dose AAV9-based desmin gene therapy is highly beneficial for the heart in DKO animals, but not in DKI mice.

## Introduction

Mutations of the human desmin (*DES*) gene on chromosome 2q35, which encodes the muscle-specific intermediate filament protein desmin, cause autosomal-dominant and autosomal-recessive forms of cardiomyopathies and myopathies [1]. As of today, more than 130 disease-causing gene alterations comprising missense, small in-frame deletion, frame shift, and splice site mutations have been reported (Human Intermediate Filament Database, www.interfil.org) [1, 2]. Cardiac involvement in desminopathies comprises hypertrophic, dilated, restrictive, and noncompaction cardiomyopathies, as well as different forms of often life-threatening cardiac conduction defects and arrhythmias [2, 3].

While the vast majority of desminopathies show an autosomal-dominant trait of inheritance and displays morphological signs of protein aggregation pathology in striated muscle, the very rare autosomal-recessive cases can meanwhile be categorized in three distinct subforms: (1) one with the complete absence of desmin protein and no obvious protein aggregation pathology [4–7], (2) one with markedly reduced protein levels of solely mutant desmin without signs of protein aggregation [8], and (3) one with markedly reduced protein levels of solely mutant desmin but the presence of protein aggregation [9–13]. In contrast to the autosomal-dominant cases, these recessive forms display a markedly more severe clinical phenotype and an earlier disease onset already starting in the first or second decade of life. Notably, the cardiac involvement determines the unfavorable prognosis with an early lethality in recessive desminopathies. No specific treatment for human desminopathies exists to date.

The desmin protein is fibrous in nature and undergoes a multistep filament assembly process, which finally leads to the formation of the three-dimensional extrasarcomeric cytoskeleton in striated muscle cells [14]. This filamentous desmin network exerts multiple roles in the alignment and anchorage of myofibrils, the positioning of mitochondria and myonuclei, mechanosensation, stress endurance, and cell signaling, and is attached to various intracellular adhesion sites by members of the spectrin superfamily such as plectin, nebulin, nebulette, and nesprin [1, 2, 15–17].

Since studies on desmin-related cardiac pathology are generally hampered by virtual absence of respective human tissue specimens, animal models for human desminopathies are valuable tools for molecular analysis and therapeutic treatment studies. In the present study, we focused on the therapeutic effect of a high-dose AAV9-mediated expression of wild-type (WT) desmin on the cardiac pathology in two autosomal-recessive desminopathy subforms. For the one with a complete lack of desmin protein we employed homozygous desmin knockout (DKO) mice [18–20], and for the one with protein aggregation pathology inflicted by the sole expression of mutant desmin we used homozygous R349P desmin knockin (DKI) mice [21]. Since previous studies showed that the human cardiac troponin T promoter enables an efficient cardiac gene expression after intravenous injections of AAV9 vectors, [22, 23] we have chosen this delivery mode also for the current study.

## Materials and methods

### Animal procedures and study protocol

**Fig. 1:**
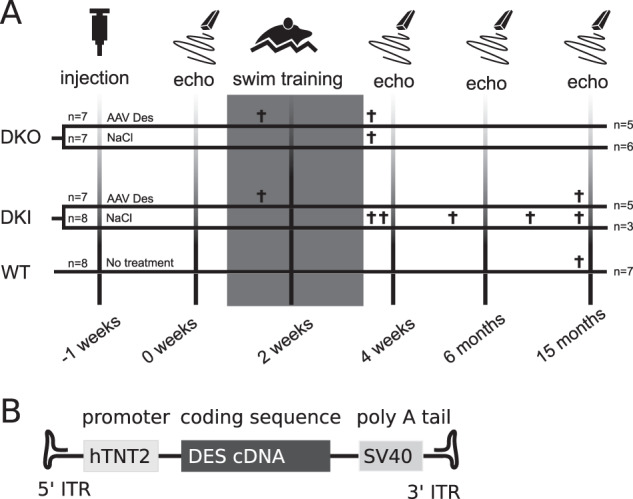
Study design. **a** Eight-week-old desmin knockout mice (DKO, *n* = 14; AAV-DES, *n* = 7; NaCl, *n* = 7) as well as homozygous R349P desmin knockin mice (DKI, *n* = 15; AAV-DES, *n* = 7; NaCl, *n* = 8) were injected with a dose of 5 × 10^12^ of AAV9-hTNT2-mDES (AAV-DES) or isotonic saline (NaCl). Eight untreated wild-type (WT) mice served as controls. Swimming experiments were performed in all groups as indicated by the gray rectangle. Mice were assessed by echocardiography (echo) prior, during and after the swimming exercise at the indicated time points after starting swimming exercise. Numbers on the right-hand and on the left-hand sides indicate the animal numbers at the beginning and the end of the experiment, respectively. Time point of death of animals that spontaneously died during the experiment are annotated by a cross. **b** The AAV-DES vector genome consists of the DES cDNA under control of the human troponin T2 ( hTNT2) promoter.

Two-month-old homozygous DKO mice (B6J.129Sv-*Des*^tm1Cba^; http://www.informatics.jax.org/allele/MGI:2159584, breeding pairs of the latter were received by courtesy from Denise Paulin, Université Pierre et Marie Curie, Paris, France) [20] and homozygous R349P DKI mice (B6J.129Sv-*Des*^tm1.1Ccrs^; http://www.informatics.jax.org/allele/MGI:5708562) [21] were treated with AAV-DES (AAV9-hTNT2-mDes, DKO *n* = 7, DKI *n* = 7) [23]; or NaCl (DKO *n* = 7, DKI *n* = 8). Randomization was performed manually shuffling cards. Sample sizes were chosen based on experiences from previous studies. A dose of 5 × 10^12^ vector genomes (VG) was systemically administered through the tail vein, resulting in a mean body weight (BW) adjusted dose of ~2.7 × 10^14^ VG/kg BW. WT littermates were used as additional controls (WT *n* = 8). A week after injection, mice were subjected to a daily 4-week program of controlled swimming exercise (see Fig. 1a) [24].

Animals were fed ad libitum and housed in a temperature- and humidity-controlled room in a specified pathogen-free environment under 12:12 h light/dark cycles. Genotyping was performed as previously described [21, 23]. All procedures involving the use and care of animals were performed according to the Directive 2010/63/EU of the European Parliament and the German animal protection code. Approval was granted by the local ethics review board (Administrative Council [Regierungspräsidium] Karlsruhe, Germany, G-143/11).

Left-ventricular function was assessed using transthoracic echocardiography before vector application, two weeks into swimming exercise, upon completion of the swimming exercise, 6 months and 15 months after vector application. All measurements were performed investigator blind. Mice were sacrificed by cervical dislocation and weighed. Hearts were removed, washed in phosphate-buffered saline (PBS), weighed and cut in half biventricularily along the SAX of the left ventricle. One half was embedded in TissueTek (Sakura, Staufen, Germany) and frozen on dry ice. The other half was further dissected. Atria and the right ventricle were removed and snap-frozen. Additional samples from liver and soleus muscle were taken. All measurements were performed by a blinded investigator.

### Generation of AAV vectors

Murine desmin cDNA was put under the control of the human troponin T promoter (Fig. 1b) and packaged in AAV9 capsids by cotransfection of pds_hTNNT2-mDES [22, 23] together with pDP9rs, a derivate from pDP2rs [25] with the AAV9 cap gene from p5E18-VD2–9 [26] in HEK293T cells using polyethylenimine (Sigma Aldrich, Taufkirchen, Germany). AAV was purified and titrated as previously described [27].

### Swimming exercise protocol

Endurance exercise was carried out as previously described [24]. Mice were subjected to a step-up swimming protocol starting with 10 min twice daily increasing the duration by 10 min each day until an exercise duration of 90 min twice daily was reached. The protocol was ended after 30 days. Mice were closely observed at all times to avoid hypoxia.

### Transthoracic echocardiography

Mice were depilated using Veet Sensitive (Reckitt Benckiser, Hull, UK). Long axis and short axis (SAX) B-mode cine loops were obtained. SAX M-mode loops were taken mid-ventricularly at the papillary muscle level. Images were obtained in hand restrained mice using the Vevo2100 system with a MS400 transducer. Three consecutive M-mode measurements of the diastolic posterial wall thickness (PWTd) were performed. Fractional shortening (FS) and left-ventricular enddiastolic diameters were obtained measuring more than 6 consecutive heartbeats using the LV-trace function on a SAX M-mode.

### Western blot

Heart tissue stored at −80 °C was thawed on ice and homogenized in lysis buffer (pH 6.8, 5 mM Tris, 10 %SDS, 0.2 M DTT, 1 mM EDTA, 100 mM NaF, 50 mM beta-glycerophosphate, 2 mM Na_3_VO_4_, 1 mM PMSF, complete mini protease inhibitor cocktail (Roche, Mannheim, Germany)). Three hundred microliters of lysis buffer was used per 10 mg of cardiac tissue. Tissue was homogenized using tissue lyser 2 (Qiagen, Hilden, Germany) and denatured by incubation at 95 °C for 5 min, upon which samples were centrifuged for 10 min at 13,000 rpm. The supernatant was aliquoted and stored at −20 °C. Lysates were adjusted to an identical total protein concentration after quantitative analysis of Coomassie-stained SDS-polyacrylamide gels. For comparative quantitative blotting, identical total protein amounts were loaded in all lanes. Gel-electrophoresis was carried out using a 12% sodium dodecyl sulfate glycine gel at 80 V for 2.5–3.0 h. Gels were blotted to nitrocellulose (Whatman Protran BA83; Sigma Aldrich) for 1.5 h with 400 mA. Membranes were blocked with milk powder (5% in TBS-T) for 1 h on a shaker and washed three times for 10 min with distilled water. Incubation with primary antibodies for desmin (DE-U-10; diluted 1:5000, Merck, Darmstadt, Germany) and GAPDH (Sigma Aldrich, 1:7500) solved in TBS-T was carried out overnight on a shaker. Secondary antibodies goat anti-mouse-HRP (Santa Cruz Biotechnology, Dallas, TX, USA) and goat-antirabbit-HRP (Santa Cruz Biotechnology) were applied for 1.5 h on a shaker at a dilution of 1:5000. Membranes were washed thoroughly in TBS-T after each antibody staining as well as after antibody stripping (Thermo Scientific, Rockford, IL, USA) which was performed between desmin and GAPDH staining. Blots were read out on a ChemiDoc XRX Imaging System (BioRad) applying Pierce ECL2 western blotting substrate (Thermo Scientific) following the manufacturer’s instructions. Expression levels were quantified using ImageJ (NIH, Bethesda, NJ, USA).

### Immunofluorescence

Frozen heart tissue embedded in optimal cutting temperature compound (TissueTek O.C.T. Compound, Sakura Finetek, Neumatten, Germany) and stored at −80 °C was cut in 9 µm slices using a Leica CM1950 cryostat and put on microscope slides. Tissues were fixed in aceton in a −20 °C freezer for 10 min, slowly thawed for ~30 min at room temperature and blocked for 1 h 10% FCS, 1% GS in PBS in a wet chamber. Slides were incubated with primary antibody RB-alpha-Desmin (Biozol, Eching, Germany, LS-B2264) diluted 1:75 in PBS and 10% FCS in a wet chamber at 4 °C overnight. Slides were washed three times for 5 min in PBS. Subsequently, the secondary antibody Alexa Fluor 488 goat anti-rabbit lgG (Thermo Fischer, Dreieich, Germany) was applied for 1 h in a wet chamber. After washing the slides three times for 5 min in PBS, cell nuclei were stained with DAPI 1:1000 in PBS, upon which slides were washed again and mounted in Mowiol mounting medium. Confocal microscopy was performed with a Zeiss LSM 780.

### Masson-Trichrome staining

Slides with heart tissue slices of 9 µm thickness were thawed at room temperature and fixed in 10% formalin for 60 min and refixed in Bouin’s solution overnight to intensify contrast and colors. The next morning, slides were washed under running tap water (18–26 °C) and nuclei were stained for 5 min with Weigert’s Hematoxylin and blued for 10 min under lukewarm running tap water to remove excess of hematoxylin, followed by a short rinse in distilled water. Cell cytoplasm, muscle, and collagen fibers were stained red for 5 min in Bieberich scarlet acid fuchsin solution. Slides were rinsed three times in distilled water for 1 min and incubated for 10 min with a combination of 1:1 phosphotungstic/phosphomolybdic acid solution, to decolorize collagen fibers of Biebrich scarlet acid fuchsin solution and prepare uptake of aniline blue. The excess solution was drained on a paper towel, upon which aniline blue solution was applied for 15 min without rinsing to stain collagen fibers. Finally, slides were washed three times in distilled water and dehydrated with graded alcohols in ascending order (70, 90, 100% EtOH) ending with Xylol for 5 min prior to mounting with Vitro-Clud mounting medium (Langenbrinck, Emmendingen, Germany). Images were acquired on an Olympus BX 51 microscope with an Olympus XC 30 camera using the cellSens software. Fibrous tissue was quantified using ImageJ as previously described [23].

### Statistics

Statistics were performed using GraphPad Prism 8 (GraphPad, San Diego, CA, USA). All data are expressed as mean ± standard error of the mean. Unless stated otherwise, a one-way-ANOVA was used to test for differences between groups. The Tukey’s honest significance test was applied for *p* value adjustment. A *p* value below 0.05 was considered significant.

## Results

Aim of our study was to investigate the long-term therapeutic effects of AAV-mediated expression of WT desmin on the cardiac pathology in two desminopathy mouse models that were exposed to strenuous exercise. As a model for the autosomal-recessive desminopathy patients lacking desmin protein expression we employed DKO mice, which display a marked cardiac phenotype [19]. As a surrogate for autosomal-recessive desminopathies with maintained desmin protein expression and the presence of protein aggregation we used homozygous R349P DKI mice as a model. The latter display cardiac pathology comprising conduction defects, arrhythmias, and a late-onset dilated cardiomyopathy [21]. We treated DKO and DKI mice with AAV9-hTNT2-mDes [23]. In order to study the long-term effect of AAV-mediated expression of WT desmin in nonsedentary animals, we subjected these mice and WT siblings to strenuous exercise employing a swimming protocol at begin of the study (Fig. 1a). We did not notice a substantial rise in mortality during the swimming experiments in contrast to a previous study reporting mortality rates of 50% in desmin deficient mice subjected to the same swimming protocol [28].

### AAV-mediated cardiac desmin expression in DKO and DKI mice

**Fig. 2:**
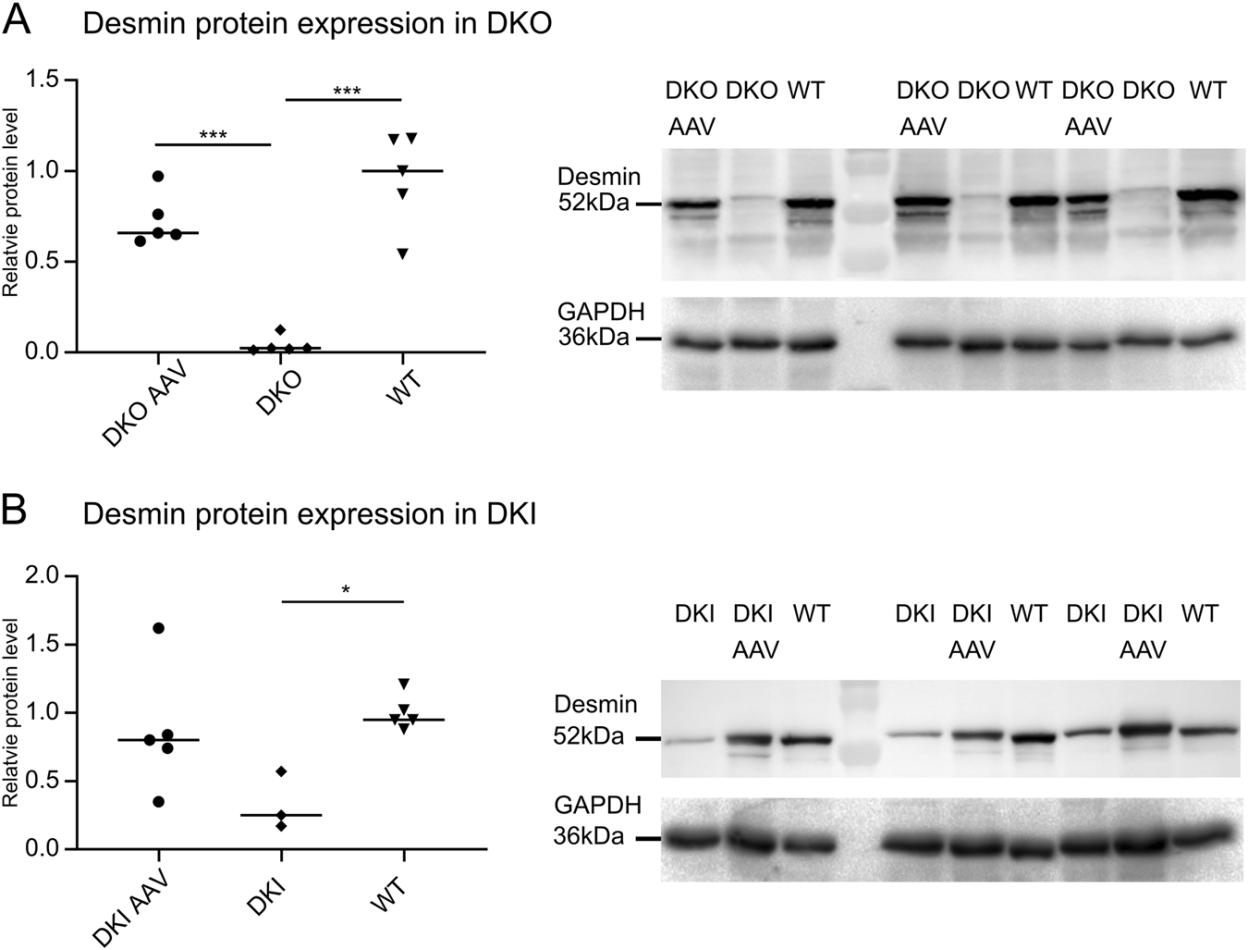
Desmin protein expression following gene therapy in DKO and DKI mice. **a** Cardiac desmin protein expression normalized to wild type in DKO mice next to a western blot stained for desmin and GAPDH. Expression levels of AAV-treated desmin knockout (DKO AAV) mice were comparable to the ones observed in wild-type (WT) mice, while no desmin expression was detectable in untreated knockout (DKO) mice. Animal numbers, DKO AAV = 5, DKO = 5, WT = 5. **b** Cardiac desmin protein expression normalized to wild type (WT) in DKI mice next to a western blot stained for desmin and GAPDH. Expression levels of AAV-treated homozygous R349P desmin knockin (DKI AAV) mice were in the same range as the ones of the wild-type (WT) mice, while untreated desmin knockin (DKI) mice exhibited significantly lower amounts of desmin. Animal numbers, DKI AAV = 5, DKI = 3, WT = 5. *p < 0.05, **p < 0.01, ***p < 0.001.

**Fig. 3:**
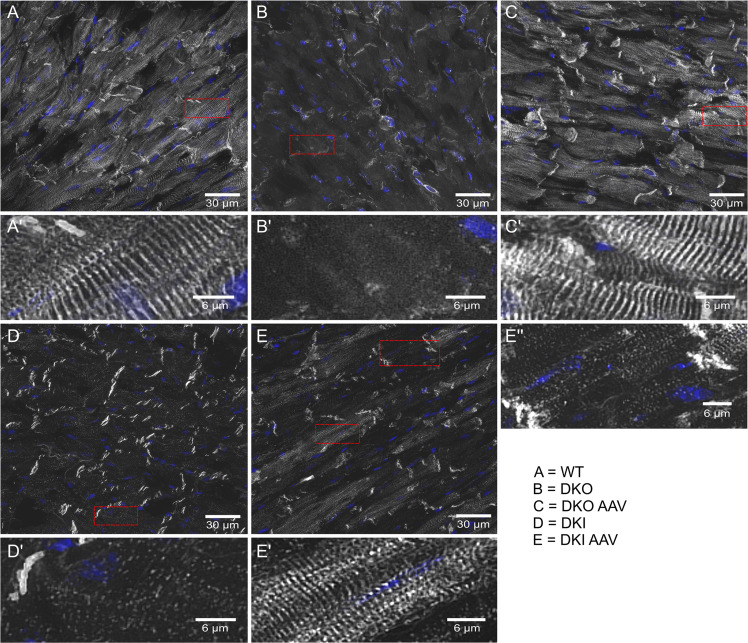
Desmin and 4′,6-diamidin-2-phenylindol (DAPI) double-staining in cardiac muscle tissue. **a** wild-type (WT), **b** desmin knockout (DKO), **c** AAV-treated DKO (DKO AAV), **d** homozygous R349P desmin knockin (DKI), and **e** AAV-treated DKI (DKI AAV); boxed areas (in red) are shown at higher magnification in (**a**′), (**b**′), (**c**′), (**d**′) as well as (**e**′) and (**e**″). The AAV-mediated expression of wild-type desmin leads to the typical myofibrillar cross striation and intercalated disc labeling patterns in the cardiomyocytes of desmin knockout mice as seen in (**c**) and (**c**′) as compared to (**b**) and (**b**′). Cardiomyocytes from DKI mice, however, showed a more complex and inhomogeneous pattern after AAV-mediated expression of wild-type desmin (**e**, **e**′, **e**″ versus **d**, **d**′). While a subset of cardiomyocytes displayed a cross striation pattern (**e**′), others only showed an intense labeling of intercalated discs in conjunction with a very weak and incomplete myofibrillar immunoreactivity (**e**″).

Desmin immunoblotting showed that vector-mediated WT desmin protein expression in AAV-treated DKO hearts reached a similar level as observed in untreated WT hearts (Fig. 2a). In hearts of homozygous DKI mice, which display significantly reduced amounts of solely mutant desmin, [21] the AAV treatment also led to higher desmin protein expression levels similar to the ones in WT animals (Fig. 2b). In order to visualize the subcellular distribution of desmin after AAV treatment, we performed desmin immunofluorescence analysis in both models. Notably, cardiomyocytes of AAV-treated DKO mice showed a desmin staining pattern that was indistinguishable from that of WT controls indicating a virtually complete reconstitution of the typical myofibrillar cross striation and intercalated disc labeling (Fig. 3a and c, c′ compare with b, b′). In contrast, analysis of the heart of AAV-treated DKI mice displayed a more complex pattern. Here, a subset of cardiomyocytes displayed a cross striation pattern (Fig. 3e′), while others only showed an intense labeling of intercalated discs in conjunction with a very weak and incomplete myofibrillar immunoreactivity (Fig. 3e, e′, e″).

### AAV-mediated expression of WT desmin rescues DKO mice from cardiomyopathy

**Fig. 4:**
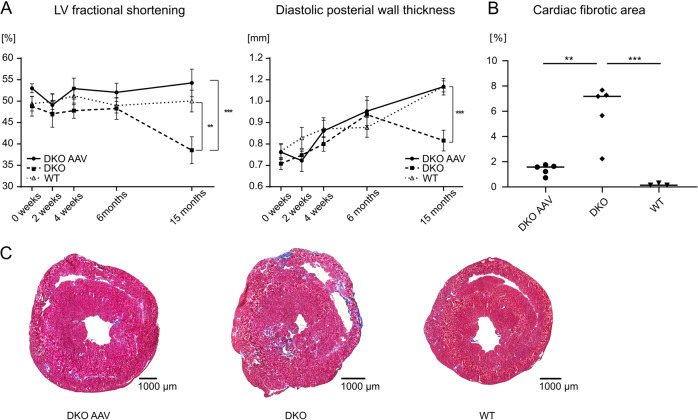
Effects of AAV-mediated gene therapy on desmin knockout mice. **a** AAV-treated knockout mice showed similar left-ventricular (LV) fractional shortening and left-ventricular posterior wall thickness as wild-type controls. Untreated DKO animals developed severe left-ventricular systolic dysfunction and thinning of the posterior wall between 6 and 15 months. Animal numbers (0 weeks/2 weeks/4 weeks/6 months/15 months) DKO AAV (7/6/5/5/5), DKO (7/7/6/6/6), WT (8/8/8/8/7). **b**, **c** AAV-mediated expression of wild-type desmin led to a significant reduction in cardiac fibrosis in DKO mice as compared with untreated controls. Animal numbers, DKO AAV = 5, DKO = 5, WT = 3. *p < 0.05, **p < 0.01, ***p < 0.001.

Mice lacking desmin develop dilated cardiomyopathy approximately starting 8 months after birth (6 months after injection, Fig. 4a) [23]. Notably, our high-dose AAV-mediated expression of WT desmin fully rescued DKO mice from developing systolic dysfunction. While untreated DKO mice showed a significant drop in FS between 6 and 15 months from 48 ± 3% to 39 ± 3% (p < 0.05, Welch’s t-test), AAV-treated DKO mice did not exhibit any significant changes in FS (6 months: 52 ± 2%, 15 months: 54 ± 3%) and showed similar values as WT animals (WT 15 months: 50 ± 3%, Fig. 4a). Furthermore, AAV treatment also significantly reduced cardiac fibrous tissue to levels close to the WT (Fig. 4b, c).

### AAV-mediated expression of WT desmin does not reduce cardiac fibrosis in DKI mice

**Fig. 5:**
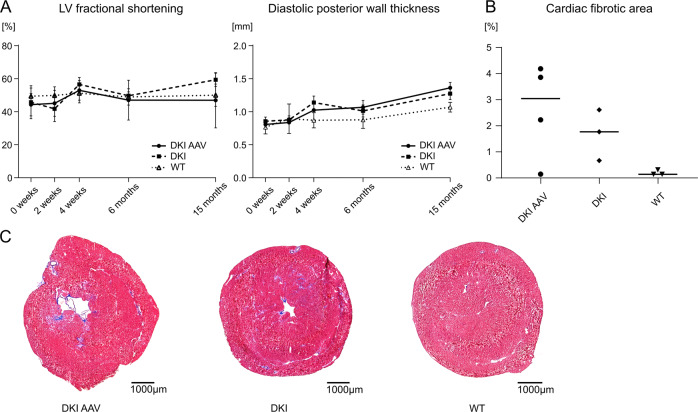
Effects of AAV-mediated gene therapy on homozygous R349P desmin knockin mice. **a** LV fractional shortening and posterior wall thickness of the left ventricle did not differ significantly between the groups. Animal numbers (0 weeks/2 weeks/4 weeks/6 months/15 months) DKI AAV (7/6/6/6/5), DKI (8/8/6/5/3), WT (8/8/8/8/7). **b,** **c** Masson-Trichrome staining revealed no statistically significant effect of AAV-mediated wild-type desmin expression on the development of cardiac fibrous tissue in homozygous R349P desmin knockin mice. Animal numbers, DKI AAV = 4, DKI = 3, WT = 3. *p < 0.05, **p < 0.01, ***p < 0.001.

A major drawback of our DKI mice, the model for the autosomal-recessive desminopathies with maintained desmin protein expression, is the late-onset and rather mild form of dilated cardiomyopathy, [21] which is highlighted by the values of the left-ventricular FS and posterior wall thickness that were in the same range as in the WT during the observation time (Fig. 5a). Thus, the effects of the AAV treatment on functional cardiac parameters cannot be sufficiently evaluated. However, though this was not statistically significant, we observed a trend toward an increase of cardiac fibrosis in AAV-treated DKI animals (Fig. 5b, c), indicating a lack of therapeutic effect.

## Discussion

### AAV9-mediated expression of WT desmin in DKO mice: a cure for the cardiac pathology

The very few thus far reported human patients suffering from a desminopathy due to a complete lack of desmin protein basically all die from their severe cardiac disease manifestation [4–7]. Since no specific therapy is currently available, we here explored the therapeutic effects of a high-dose AAV9-based expression of WT desmin in the hearts of DKO mice. In a previous study, we already demonstrated that a dose of 3 × 10^12^ VG led to a low-level cardiac re-expression of desmin (20% as compared with the WT) and an amelioration of the morphological and functional cardiac pathology in the treated DKO mice [23]. By further increasing the dose to 5 × 10^12^ VG, we were now able to fully normalize the level of desmin protein in cardiac tissue of the treated animals. On the morphological level, this was associated with the complete reconstitution of the three-dimensional desmin network in virtually all cardiomyocytes. Notably, this successful restoration of the intermediate filament network on the cellular level not only reversed the degree of cardiac tissue fibrosis, but also led to a complete normalization of the functional cardiac parameters, namely the left-ventricular FS and left-ventricular posterior wall thickness. Thus, the current study shows that increasing the AAV dose results in an improved therapeutic effect in DKO mice.

A previous study reported that strenuous physical exercise led to a mortality rate of up to 50% in DKO mice [28]. This, however, could not be reproduced in the present study, which employed the same swimming protocol. Out of seven AAV DKO and seven NaCl DKO mice, only two and one, respectively, died during the 15-month course of this study. Here, it is tempting to speculate that the discrepancy might result from different genetic backgrounds. DKO mice of the above-mentioned study had a 129 Sv background, while our DKO mice were in a C57BL/6J background [29]. Moreover, even different sub-strains of C57BL/6J have been reported to diverge in their susceptibility to pressure overload induced heart failure [30]. Our discrepant findings might be traced back to the fact that C57BL/6J mice seem to be more resilient to pressure overload and oxidative stress due to a deletion in the Nnt gene coding for the mitochondrial NAD(P) transhydrogenase [31].

Translated into the human disease context, our findings in DKO mice strongly indicate that an AAV9-based gene therapy approach, even at a single time point, may serve as a long-term therapeutic measure for this specific group of desminopathy patients. However, a further clinical translation will require dose finding studies in a large animal model and finally patients in order to identify the lowest effective dose.

### Desmin gene therapy in DKI mice: no clear benefit for the heart

To date no mouse model exists, which truly mirrors the genetic alterations in human autosomal-recessive desminopathies with maintained desmin protein expression. As a surrogate, we used our homozygous R349P DKI mice, which show desmin protein aggregate pathology as well as a cardiac phenotype comprising conduction defects, arrhythmias, and a late-onset dilated cardiomyopathy [21].

In untreated mice of this model, the desmin protein level—solely mutant desmin—in cardiac tissue is ~25% of the desmin amount in WT animals [21]. In analogy to the treated DKO mice, our gene therapy approach in the homozygous DKI mice also resulted in seemingly normalized total desmin protein amounts. However, on the morphological level only a subset of cardiomyocytes displayed a normal desmin cross-striated staining pattern indicating that the individual, cell-specific mixture of WT, and mutant desmin influences the stability of the desmin intermediate filament network and the turnover of the different desmin protein species. This notion is in line with our previous results, in which WT and mutant desmin proteins displayed different patterns of turnover in skeletal muscle tissue of heterozygous and homozygous R349P DKI mice and WT littermates [21]. Notably, the increased desmin protein levels were also associated with a trend toward an increase of cardiac fibrosis in AAV-treated DKI animals, which might reflect a harmful effect on cardiac tissue in this setting where both WT and mutant desmin proteins are present.

Out of seven AAV DKI and eight NaCl DKI mice a number of two and five animals, respectively, died during the course of this study. However, the applied 4-week protocol of strenuous exercise did not accelerate the development of cardiomyopathy in DKI mice during the long-term follow-up for 15 months after vector injection. Thus, impaired left-ventricular function can be ruled out as potential cause. Previous electrophysiological investigation in R349P DKI mice during swimming exercise documented an increased number of premature ventricular contractions and higher grade atrio-ventricular and sinu-atrial blocks compared with WT controls [21]. However, since there was no difference in electrophysiological parameters between homozygous and heterozygous R349P DKI mice in our previous study, we did not consider potential effects of desmin-overexpression on electrophysiology and have thus not performed Holter-EKGs [21]. Although the overexpression of WT desmin had no beneficial effect on cardiac fibrosis of DKI mice, further electrophysiological studies are necessary to investigate whether the trend toward increased mortality in control-treated DKI mice can be explained by sudden cardiac death induced by malignant arrhythmias or conduction defects.

Taken together, in contrast to DKO mice, in which the expression of WT desmin resulted in a significant reduction of cardiac fibrosis and protection from heart failure, the cardiac phenotype of DKI mice, i.e., the extent of cardiac fibrous tissue, could not be ameliorated by gene transfer of WT desmin. Thus, future gene therapy approaches for the cure of cardiomyopathy in desminopathies that are based on the overexpression of WT desmin appear particularly useful for patients with a complete lack of desmin protein.
